# The contagion of neurologic Immersion predicts retail purchases

**DOI:** 10.3389/fnins.2025.1533784

**Published:** 2025-03-12

**Authors:** Gaia Rancati, Kankana Ghosh, Jorge Barraza, Paul J. Zak

**Affiliations:** ^1^Department of Marketing, Middle Tennessee State University, Murfreesboro, TN, United States; ^2^Department of Computer Science, University of Bologna, Bologna, Italy; ^3^Department of Psychology, University of Southern California, Los Angeles, CA, United States; ^4^Center for Neuroeconomics Studies, Drucker School of Management, Claremont Graduate University, Claremont, CA, United States

**Keywords:** neurophysiology, synchrony, prediction, field experiment, consumer behavior

## Abstract

Consumers increasingly demand extraordinary experiences and businesses want to provide such experiences to build loyalty and increase customer lifetime value. One of the most significant aspects of consumer experiences is employee-customer interactions. We hypothesized that the value of customers’ experiences would be reflected in the neurophysiology of sales associates and that these data would predict eventual purchases. We tested this hypothesis by measuring neurologic Immersion of sales associates serving customers (*N* = 56) in a field study in two luxury retail stores with actual customers. A synthetic dataset was generated from these data and showed that sales associates’ peak Immersion was positively associated with the time customers spent shopping, which, in turn, positively scaled with how much customers spent. Estimating a machine learning model using sales associates’ peak Immersion predicted which customers purchased with between 64% and 80% accuracy. Our results demonstrate that the neurophysiologic Immersion of one person can be used to predict the behavior of another person with whom they are interacting even when their goals may not be perfectly aligned. Moreover, we have shown that such a field study is feasible with real customers who are spending nontrivial amounts of money (M = $323, range: $0–$2,734). More generally, measuring the contagion of Immersion from one side of an interaction may be an effective way to assess and improve the quality of social engagements of many types.

## Introduction

The retail industry is undergoing a significant transformation with an increased focus on customer experience. This change is particularly evident in the luxury segment that is experiencing rapid growth (Back et al., 2022). The global luxury market will generate $238.5 billion in sales by 2028, up from $93.4 billion in 2022, fueled by a surge in consumer demand for luxury experiences (D'Arpizio et al., 2021; Bell, 2022; Fortune Business Insights, 2024). Luxury brands can only reach these heights by enhancing their value to consumers through extraordinary and personalized customer experiences (Kapferer, 2015).

Luxury products and services transcend pure functionality by communicating prestige, exclusivity, and exceptional quality (Kapferer, 2012; Wirtz et al., 2020; Berry and Bendapudi, 2003; Moore and Doherty, 2007; Ko et al., 2019). During service encounters, sales associates in luxury stores typically follow a crafted script, known as the personal selling process, to ensure that a quality experience is delivered (Dubinsky, 1981; Solomon et al., 1985). The selling script is designed to add experiential value to the purchased product or service (Cervellon and Coudriet, 2013; Pernice, 2016; Dion and Borraz, 2017). Customer service scripts increase customer attention (Jacobs, 2003; Lent and Tour, 2009), boost engagement (Carù and Cova, 2006), and increase in-store dwell time (Rancati and Maggioni, 2023). They are also meant to increase customer lifetime value by inculcating loyalty (Cambra-Fierro et al., 2014; Nguyen et al., 2014). While retailers seek to provide meaningful customer experiences, the extant research has predominantly focused on capturing self-reported customer perspectives while ignoring direct data from sales personnel (Carù and Cova, 2006; Frei et al., 2012; Pernice, 2016).

Compelling service interactions synchronize the experiences of sales associates and customers (Macintosh, 2009). Synchronicity improves the perception of sales associates’ skills and abilities (Gremler et al., 2001a, 2001b), trust (Doney and Cannon, 1997), and the enjoyment of interactions (Macintosh, 2009). For example, synchronicity can be obtained by sharing a narrative about the brand with customers (Green and Brock, 2000; Mainemelis, 2001; Bracken et al., 2014). When customers are attentive and emotionally involved in the experience, they become part of the story itself (Green and Brock, 2000; Baek and Morimoto, 2012; Gerrig, 2018). In luxury stores, narrative cues are woven into the service script in which sales associates serve as brand ambassadors (Kingman-Brundage, 1989; Smith, 2016; Rancati and Maggioni, 2023; Abelson, 1981; Nguyen et al., 2014; Jacobs, 2003; Cervellon and Coudriet, 2013).

The present research investigates whether sales associates’ neurophysiological responses sufficiently reflect customers’ experiences such that the former predicts the purchase behavior of the latter. If our hypothesis is supported, we will have provided a feasible and accurate way to quantify the value of the employee-customer interactions without interrupting the sales process, relying on inaccurate retrospective reports, or seeking to infer best practices from ex-post sales data (Darley et al., 2010; De Keyser et al., 2019). These traditional methods are influenced by situational and cultural contexts (Zhao et al., 2018), physiological states (Christensen et al., 2011), socioeconomic status (Fazio and Olson, 2003), poor recall of events (Furnham, 1986), and social desirability (Fisher and Katz, 2000). Furthermore, self-report measures poorly capture the dynamic nature of service interactions (Nguyen et al., 2014; Verhulst et al., 2020). Recent research has emphasized the need for more objective measurement methods in this area (Benoit et al., 2017; Verhulst et al., 2019), with neurophysiologic data proving particularly valuable in measuring customer behavior (Plassmann et al., 2015; Spence, 2019), in-store customer experiences (Rancati et al., 2024), and service scripts (Rancati and Maggioni, 2023).

Our approach was motivated by an extensive set of findings showing synchronized physiologic responses by individuals during shared experiences (Lindenberger et al., 2009; Holper et al., 2012). For example, neural synchrony occurs when children listen to a story being read to them (Piazza et al., 2021). Yet, most techniques to measure neurophysiologic synchrony, such as functional magnetic resonance imaging or electroencephalograms, are impossible during natural interactions and thus are inappropriate for a field study to measure encounters with actual customers shopping in an actual store.

### Neurophysiologic Immersion

Our goal is to accurately predict customer behavior from sales associate neural responses. There are a variety of physiologic signals that could be measured in a field study, but most of them are not optimized to predict behavior and generally do so poorly (Zak, 2022). In 2015, the US Defense Advanced Research Projects Agency (DARPA) initiated a program called Narrative Networks to identify combinations of neurophysiologic signals that would accurately and consistently predict behaviors after a message or experience (Casebeer, 2018). As part of this research team, our group measured approximately 140 signals simultaneously from the central and peripheral nervous systems in a series of experiments over several years in which participants had a free choice to take an observable action or not (Barraza and Zak, 2009; Zak, 2022). Neural activity for responders versus non-responders was contrasted and as experimental data accumulated, signals that failed to increase predictive accuracy or were redundant were eliminated.

Neurophysiologic Immersion combines neural signals associated with attention and emotional resonance to predict behavior (Barraza and Zak, 2009; Zak and Barraza, 2018; Zak, 2022). Immersion appears to capture the value associated with social–emotional experiences (Zak, 2022) and was developed explicitly to predict behavior (Lin et al., 2022; Barraza and Zak, 2009). The commercial platform used to measure Immersion applies algorithms from signals in the peripheral nervous system derived from variations in cardiac rhythms which are convolved to maximize predictive accuracy, producing a single 1 Hz data stream (Zak, 2020). The platform has been in commercial use since 2017 by companies such as Accenture, advertising agencies, movie studios, TV networks, as well as research laboratories (Zak, 2022; Accenture, 2018). Since Immersion was designed to predict behaviors, it is an attractive candidate to capture neurophysiologic contagion that could predict customer actions. Moreover, these data can be collected by unobtrusive arm-worn sensors sending data to a commercial platform, so it is well-adapted to a field study where the sales associates do not want to bother, or appear odd, to customers in a luxury retail setting who are expected to make large purchases.

Neurologic Immersion has been shown to accurately predict both individual and population outcomes with 83–97% accuracy. For example, Immersion accurately identified which videos people choose to watch (Lin et al., 2022), which new music became hit songs (Merritt et al., 2023), how much people enjoyed theatrical performances (Melton et al., 2024), which individuals volunteered to support environmental protection (Morris et al., 2019), and how people responded to retail assistant robots (Rancati and Maggioni, 2023). Immersion also correctly classified mood and energy with 98% accuracy (Merritt et al., 2023). This approach has been called “brain as predictor” (Falk et al., 2012; Berkman and Falk, 2013).

### Model and hypotheses

We built a mathematical model to identify how neurologic responses during retail shopping might influence choices. This model was developed to generate empirically testable hypotheses because there is very little extant theory on neurophysiologic contagion during consumer choices and none that used neurophysiologic data from a sales associate to predict the behavior of a shopper (Herrando et al., 2022; Rosenbaum et al., 2021). The model starts with a consumer who has a choice to shop at store 1, purchasing a vector of goods *c_1_* or store 2 purchasing vector *c_2_*. Prices in each store are the same, *p_1_* = *p_2_* = *p*, but store 1 includes a utility flow from the shopping experience, *e*. In order to make the predictions concrete, we use a logarithmic utility function.


$$\max_{c1,c2}\;e\left[\ln\left(c_{1}\right)\right]+\ln\left(c_{2}\right)$$

$$s.t.\;p\;c_{1+}\;p\;c_{2}<M$$

where *e* is the value obtained from the shopping experience in store 1 that depends on the experiential value one gets from shopping *e > 1*, and *M* is income. This is a standard two-good utility maximization model from economics (Mas-Colell et al., 1995) to which we have made a single addition, the experiential value of shopping (*e*). This parameter was included to explore how the process of shopping could affect choices. Models of this class have been called procedural utility as they include a utility flow from both consumption and the process of obtaining consumer goods (Frey et al., 2004).

The constraint in the optimization model restricts the consumer from spending more than his or her income (credit is not included in the model for simplicity). The “procedural” component of this model is the utility of the shopping experience, *e*, that depends on the neurologic value of the experience *e*(*Im*), where *Im* denotes Immersion and *e'(Im)* > 0. The model shows that, all else being equal, shoppers will prefer purchasing from store 1 because the shopping experience is valuable to the customer (Pine and Gilmore, 2019).

The model can be solved by substituting the constraints into the objective function, differentiating it, and setting it equal to zero to find the maximum (Mas-Colell et al., 1995). The optimal purchase amount for store 1, c_1_*, in which the shopping is enjoyable, is


$$pc_{1=}^{*}\frac{\mathrm{Me}}{p+e}\text{.}$$

We impose the assumption that the value of the shopping experience is proportional to the time spent shopping, *e(Im)* ∝ *t.* That is, a customer who is not receiving sufficient neurologic value from shopping will leave the store sooner than will a customer for whom the experience is better.

The model predicts that,

1. Customers spend more money in stores that provide a valued experience, pc_1_* > pc_2_*,
2. Consumers will extend the time spent shopping (*t*) when the neurologic value of the shopping experience *e*(*Im*) increases;
3. The purchase amount in a store that provides an experience while shopping, pc_1_*, is increasing in the value obtained from the experience, *e*(*Im*).

An experiment was designed and run to test these hypotheses.

## Methods

### Participants

This study was approved by the Institutional Review Board of Claremont Graduate University (#3384). The owners of two high-end retail stores in California gave the researchers permission to conduct the study on their premises. One store sold women’s clothing (WS), and the other sold men’s clothing (MS). Sales associates were introduced to the study’s goals and the neurophysiologic sensors’ functionalities. One sales associate from each store provided written informed consent to having data collected during a four-week study with their identities masked in the dataset. In order to reduce noise in the data, neurophysiologic data from the same salesperson were collected throughout the study. The sales associates at both stores were similar: they had 20 years of experience in clothing sales, were female, and were between 50 and 55 years old.

Data collection began when sale associates welcomed customers. Then, sales personnel presented the store and the merchandise, offered advice, responded to customers’ requests, and completed the sale. The study did not inhibit customers from browsing the store and no maximum duration was imposed on the shopping experience. Each customer interaction was video recorded and timed to measure visit duration by on-site researchers who were not visible to participants (Tunnell, 1977; Crano et al., 2014; Rancati and Maggioni, 2023). Data were collected on Thursdays, Fridays, and Saturdays when foot traffic in both stores was highest. Customers from whom purchase data were collected provided written informed consent after paying for their purchases. Customers were informed that their data from would be anonymized in the dataset, and they could refuse to participate if they so choose. Post-data collection consent is ethically acceptable (Speer and Stokoe, 2014) and no customers refused. A total of 56 customers were included in the study with similar age distributions across stores (WS: M = 48.83, SD = 11.88; MS: M = 51.0, SD = 13.5).

### Neurophysiology

Neurologic responses were obtained using a commercial neuroscience as a service (NaaS) platform called Immersion (Immersion Neuroscience, Henderson, NV). Sales associates were fitted with Scosche Rhythm+ wearables (Scosche Industries, Oxnard, CA) that included photoplethysmography (*PPG*) sensors. PPG is a non-invasive optical technique used to detect volumetric changes in blood in peripheral circulation using a light emitting diode (LED) and software that identifies the cardiac cycle (Nitzan and Ovadia-Blechman, 2022). Associates were advised to wear sensors moderately tightly on their nondominant forearms in order to reduce signal loss from lack of skin contact. Cardiac data were sent to cloud servers where the Immersion Neuroscience platform uses algorithms to derive neural responses from the cranial nerves (Zak, 2022; Barraza and Zak, 2009). The platform provides an output file of cleaned, signal-processed data used for analysis. Neurologic Immersion is well-suited for field studies as we performed here because it is inconspicuous, and the data are motion-corrected (Zak, 2022). No native signals from wearables were used in the analysis. The analysis used both average Immersion of the sales associate while they assisted a customer as well as a derived variable called Peak Immersion following previous research (Merritt et al., 2022),


$${\mathrm{Peak Immersion}}_{i}=\frac{1}{I_{i}}\overset{T}{\underset{t=0}{\int }}\left(n_{ijt}>M_{i}\right)d_{t}$$

where $n_{ijt}$ is neurophysiologic Immersion for sales associate j at time t when first encountering customer *i* at *t = 0* until the customer left the store at $T,\;M_{i}$ is the median value of Immersion while shopper *i* is in the store plus 0.5 standard deviation of Immersion, and *I_i_* is the total Immersion while person *i* is shopping. More simply, Peak Immersion cumulates the highest Immersion parts of each shopping experience by adding up the peaks of Immersion above the threshold $M_{i}$ and normalizing it by total Immersion to control for differences in the time spent shopping (dwell time). The brain has a strong tendency to return to basal activity and for long data collections peak Immersion tends to be a more accurate predictor of behavior than average Immersion (Merritt et al., 2023). While purchases in both stores were similar, the shoppers were not (see Results).

### Statistical analysis

We used a sequence of statistical approaches, increasing in sophistication, to assess the ability of sales associate Immersion to predict customer purchases. The analysis begins with tests of mean differences for demographic and neurophysiologic data comparing the WS and MS using Student’s *t*-tests and Chi-squared tests to compare proportions. Parametric relationships were examined using Pearson correlations while logistic regressions were estimated to establish predictive accuracy. Since our goal is to predict customer behavior using Immersion measured in sales associates, and, as shown below, the behavior of shoppers in the two stores was different, separate analyses and predictive models are estimated for each store.

### Synthetic data

Due to the moderate sample size, a synthetic dataset was generated by utilizing the R package *synthpop*, employing the *syn()* function applied to the original dataset. This standard procedure creates observations by repeatedly randomly sampling the joint distribution of the data. This technique is used when obtaining large datasets is infeasible, including analyses of computer vision (Mayer et al., 2018), sensitive information like hospital records (Tucker et al., 2020), and with unbalanced data (He et al., 2008; Luo et al., 2019). The parameters used for synthetic data generation were standard, including method (parametric), minimum levels (1), and seed (1969). The number of observations was set to 10,000. The supplementary material compares the observed data to the synthetic and shows they are statistical matches (Supplementary material Figures A1–A6).

### Mediation model

We constructed a mediation model to test the causal relationship shown in the mathematical model between Immersion, dwell time, and purchases (Agler and De Boeck, 2017). The model was estimated using maximum likelihood using the R package *NLMINB*.

### Machine learning

After performing traditional statistical analyses, we harnessed the capabilities of machine learning (ML) to build predictive models of purchases. The synthetic data were modeled using an ensemble classification approach, leveraging the strengths of three distinct algorithms: random forest, XGBoost, and CatBoost. Ensemble ML flexibly combines multiple ML models to improve training and predictive accuracy over that of a single ML model (Dietterich, 2000). Random forest is robust and able to handle noisy data, making it an excellent choice for neurophysiologic data. The second model type, XGBoost, improves the performance of weak learners, enhancing predictive accuracy. Lastly, CatBoost, with its categorical feature support and optimized training, was expected to contribute to the ensemble’s overall robustness. One-half of the synthetic data was used to train the ensemble ML model and tune the hyperparameters. The other half of the synthetic data was used to test the models.

To ensure the reliability of our findings, we subjected the ensemble model to a rigorous cross-validation process, dividing the dataset into five folds. This approach allowed us to assess the model’s performance across various subsets of the data, ensuring its generalization capabilities and reducing the likelihood of overfitting. Model performance included sensitivity (true positive rate), specificity (true negative rate), area under the curve (AUC), and mean squared error (MSE). Analyses were done with neural variables alone and with the inclusion of the control variables *loyal* (the customer had made previous purchases from the store) and *companion* (the shopper brought a companion along).

### Data availability statement

The data are available at Open ICPSR openicpsr-203881.

## Results

### Behavior

The women’s and men’s stores had different customer profiles, each with distinct characteristics and behavior. The WS predominantly attracted female customers (*N* = 31, 90% female, age: M = 48.83, SD = 11.88), while the MS had fewer female customers (*N* = 25, 52% female, age: M = 51.0, SD = 13.5). Customers in the WS spent on average twice as long shopping (minutes) compared to customers in the MS (WS: M = 32.15, SD = 35.37; MS: M = 14.69, SD = 10.93; t = 2.372, *p* = 0.0213). The longer shopping time was reflected in both the frequency of purchases (WS: 0.73; MS: 0.52; χ^2^ = 0.127, *p* = 0.722) and the amount of money spent (WS: M = $265.79, SD = $517.37; MS: M = $140.12, SD = $229.42; t = 1.125, *p* = 0.266). Indeed, the maximum spent in the WS was more than 200% above the maximum in the MS (WS: $2,734.22; MS: $810.00). Shoppers in both stores were equally likely to be return customers (WS: 43%; MS: 52%; χ^2^ = 1.9439, *p* = 0.1632).

### Neurophysiology

**Figure 1:**
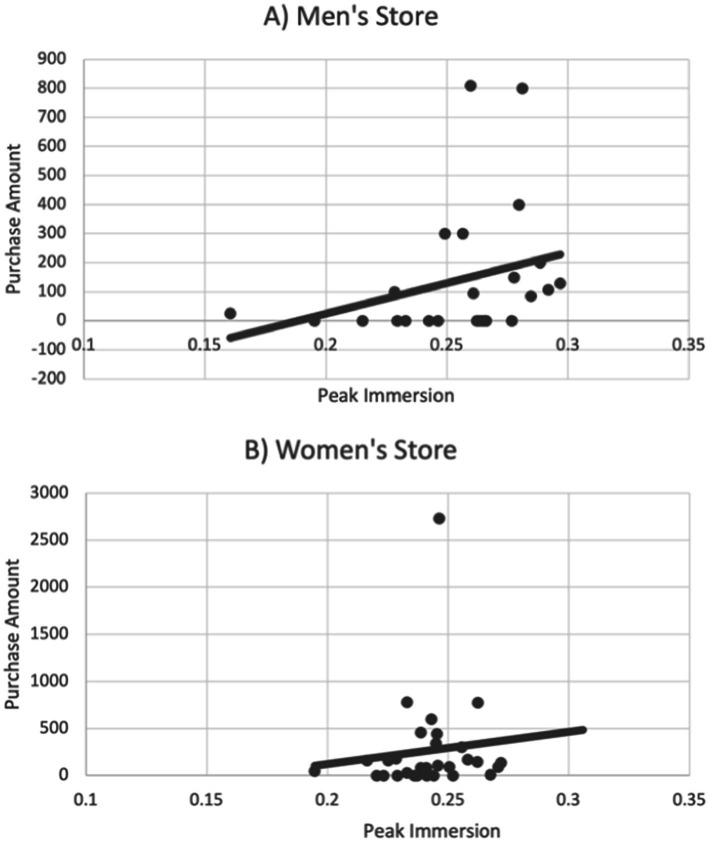
Average peak Immersion for sales associates in **(A)** the men’s store trended toward significance in its association with customer purchase amount (*p* = 0.07) without taking in account customer dwell time. The positive relationship did not hold in **(B)** the women’s store (*p* = 0.28).

The behavioral differences in each store were reflected in different neurophysiologic responses. Average Immersion did not differ by store (WS: M = 4.422, SD = 0.214; MS: M = 4.486, SD = 0.240; t = −1.044, *p* = 0.301), but Peak Immersion was higher in the men’s store (WS: M = 0.2418, SD = 0.017; MS: M = 0.255, SD = 0.032; t = −1.974, *p* = 0.05). The correlation between average peak Immersion for sales personnel and purchases by customers in the men’s store was positive and trended toward significance even without taking in account customer dwell time (MS: r = 0.29, one-tailed t = 1.46, *p* = 0.07). The relationship between peak Immersion and purchases had the correct sign but was not significant for the women’s store (WS: r = 0.11, one-tailed t = 0.60, *p* = 0.28; Figure 1).

### Time

The time spent shopping and purchase amounts were correlated in the MS but not the WS (WS: r = 0.253, *p* = 0.177; MS: r = 0.536, *p* = 0.005). Yet, in both the WS and MS the correlation between average Immersion and time spent shopping, as predicted by the mathematical model, failed to be significant (WS: r = −0.112, *p* = 0.553, MS: r = 0.033, *p* = 0.875). The relationship with time also failed to be significant for Peak Immersion (WS: r = 0.219, *p* = 0.244, MS: r = 0.347, *p* = 0.088).

### Synthetic data

The insignificant findings with trends consistent with the mathematical model may be driven by the small sample size based on the logistical constraints imposed on the study in order to collect an ecologically valid sample. In order to examine if the results that trended toward significance were due to the moderate sample size, we generated a synthetic dataset to further test the relationship between Immersion, time, and purchases. The synthetic data closely resembles the original data. The mean squared errors (MSE) of the differences between the synthetic and observed data were minimal (WS: *N* = 10,000, MSE: M = 1.818182e-07, SD = 4.045199e-07, MS: *N* = 10,000, MSE: M = 9.090909e-08, SD = 3.015113e-07). Similarly, the two datasets’ distributions were statistically equivalent using the Kolmogorov–Smirnov (KS) test (WS: *p* = 0.977; MS: *p* = 0.996).

### Synthetic data analysis

The synthetic data show Immersion was significantly higher in the MS compared to the WS though the size effect was small (WS: M = 4.422, SD = 0.210; MS: M = 4.483, SD = 0.234; t = −19.066, *p* = 0.000). Similarly, Peak Immersion was higher in the men’s store (WS: M = 0.242, SD = 0.017; MS: M = 0.254, SD = 0.032; t = −34.746, *p* = 0.000). The synthetic data confirmed the positive relationship between the time spent shopping and purchase amounts in both stores (WS: r = 0.153, *p* = 0.000, MS: r = 0.414, *p* = 0.000). The larger dataset captured the positive statistical association between Peak Immersion and time spent shopping in the stores (WS: r = 0.318, *p* = 0.000 r = 0.345, *p* = 0.000). Average Immersion was also associated with time shopping but carried the wrong sign for the WS and its correlation was smaller than for Peak Immersion (WS: r = −0.184, *p* = 0.000, MS: r = 0.129, *p* = 0.000). As a result, the following analyses focus on Peak Immersion as the neurophysiologic measure of the value of the shopping experience.

### Mediating effects of time

**Figure 2:**
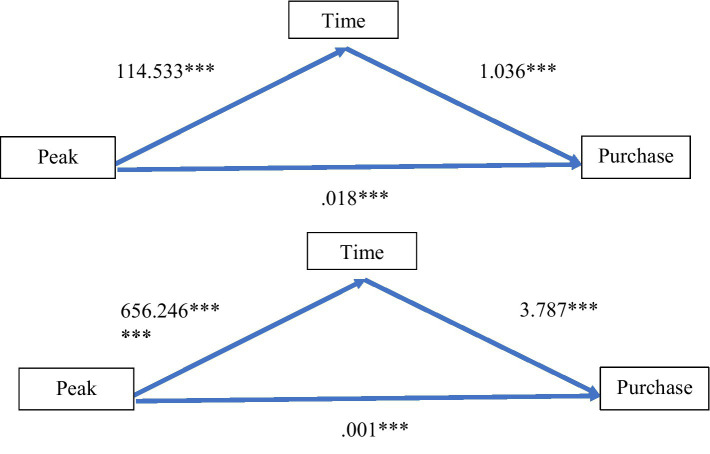
The mediating effects of Peak Immersion on time and purchases depicting the coefficients for both paths. The top panel is the women’s store and the bottom panel is the men’s store. The upward sloping coefficient captures the relationship from Peak Immersion to Time, while the downward sloping coefficient is the Time to Purchase association. The horizontal coefficient is the direct effect of Peak Immersion on Purchases. ***indicates a *p <* 001 showing that both the direct and indirect relationships are statistically significant.

A mediation model was estimated using the synthetic data to examine the direct and indirect effect of Peak Immersion on purchases for each store separately. The mediation model showed that Peak Immersion directly influenced the time spent shopping in both the MS and WS. Further, as predicted, Peak Immersion mediated the purchase decision by increasing the time spent shopping (Figure 2). Goodness of fit measures for the model were in acceptable ranges (Supplementary material Table A1). The results continued to hold when covariates *loyal* and *companion* were included in the analysis (Supplementary material Table A2). The mediation model was also estimated using the original data, showing that the mediation by time continued to hold for the MS, even in this restricted sample, but failed to be significant for the WS (Supplementary material Tables A3, A4).

### Predicting purchases

After discretizing the purchase variable, an ensemble classification model was estimated using Random Forest, XGBoost, and CatBoost models including Peak Immersion and time as independent variables, with controls *loyal* and *companion*. The hyperparameters used for the ensemble model included: RandomForestRegressor (n_estimators, random_state), XGBRegressor (learning_rate, max_depth, n_estimators, random_state), CatBoostRegressor: (silent, random_state). The optimal hyperparameters for these models were found by creating a dictionary where each key is a hyperparameter name, and the corresponding value is a list of hyperparameter values to be tested. Subsequently, the *Scikit-learn* function *GridSearchCV* evaluated different combinations of hyperparameters while performing cross-validation. This hyperparameter tuning process was performed separately for each model.

The ensemble model performed moderately well on sensitivity for the MS and very well for the WS (MS: 77.3%, WS: 97.6%; AUC = 0.666) but poorly for specificity (MS: 48.99%, WS: 4.4%; AUC = 0.707). Overall accuracy was 80% for the WS and 64% for the MS. A five-fold cross-validation showed the model was not overfit (MS: MSE = 0.2235; WS: MSE = 0.156).

## Discussion

The findings reported here extend the extant literature in several key ways. First, we showed that the neurophysiologic activity of one person accurately predicts the behavior of another person. Synchrony typically arises in setting of shared experiences when people communicate, move together, or listen to music (Keller et al., 2014; Stevens et al., 2009; Samadani et al., 2021; Semin, 2007; Stuldreher et al., 2020). We hypothesized that neurophysiologic synchrony would arise in a retail shopping setting, but rather than simply show synchrony between sales associates and customers, we sought to predict customer purchases using the neurophysiologic data from salespeople. This adds a new finding to the study of synchrony. We showed that the contagion of neurophysiologic signals predicted which customers would make purchases with 64–80% accuracy, and the purchase amount was positively mediated by the time customers spent shopping. Our finding that customer dwell time increased purchases in a high-end retail setting is consistent with previous findings (Mihić et al., 2018; Li et al., 2021). Additionally, our analysis showed that customers’ time spent shopping increased linearly with sales associate peak neurologic Immersion, providing a mechanism through which dwell time can be influenced. Related research has shown that neurologic Immersion predicted how long people watched videos about social ills and that viewing time increased the likelihood that participants would donate to a charity that addressed such ills (Lin et al., 2022).

Our second key finding contributes to literature on mentalizing about others’ intentions, often measured using an electroencephalogram, to the use of the omnibus neurophysiologic measure Immersion. Immersion was developed to accurately predict individual behaviors (Zak, 2022; Merritt et al., 2022) and has also been shown to predict population behaviors (Merritt et al., 2023; Zak, 2022). The ability of Immersion to accurately explain the action of person A by measuring person B indicates the value of using multiple convolved neural measures such as Immersion. This finding is moderately surprising since sales associates and customers are on different sides of a transaction, though both presumably desire their interaction to be valuable and enjoyable (Haas and Kenning, 2014; Kim and Kim, 2014; Lee and Dubinsky, 2003). Nevertheless, sales associates and customers may have at least partially different goals for their interactions that would be expected to reduce cross-individual predictive accuracy. Yet, not only was predictive accuracy high, but purchase amount scaled linearly with salesperson Peak Immersion. Since interpersonal synchrony facilitates memory encoding, this finding suggests that Peak Immersion experiences may increase customer loyalty by making the shopping experience more memorable (Hasson et al., 2008).

Third, the data were collected in a field study using wearables and a commercial neuroscience as a service (NaaS) platform. As a result, this study is ecologically valid (Smith, 2003; Kihlstrom, 2021) as it measured salespeople and customers in the setting in which they naturally meet. The quantitatively higher values for average dwell time and purchase amount at the women’s store compared to the men’s store show the importance of using actual customers rather than study participants who may be given specific tasks during a limited period of time (El Hedhli et al., 2016; Horváth and Adıgüzel, 2018; Kotzé et al., 2012). But, at the same time, Peak Immersion was higher in the men’s store in which one-half of the shoppers were women. This suggests that shopping for someone other than oneself may be more valuable neurologically, even if the average time spent shopping in the men’s store was less than that in the women’s store (Gillison and Reynolds, 2016).

Fourth, an innovation in our work was to estimate both a mediation model to examine the effect of Peak Immersion on dwell time and purchases, as well as estimating an ensemble ML model to quantify the predictive accuracy of these variables. The mediation of time was predicted by the mathematical model we presented and confirmed in our analyses. The ML model was estimated because it effectively captures the inherent nonlinearities in neurophysiologic data, improving predictive accuracy relative to (log) linear models such as logistic regressions (He and Yang, 2021; Merritt et al., 2023).

Lastly, our findings have implications for measuring Immersion in a service context. By showing that Immersion of sales personnel accurately reflects the emotional experience of customers, our study provides managers with a methodology to create more valuable shopping experiences that could be customized for various customer segments. Although the study focused on high-end retail, organizations could measure Immersion during each stage of the service encounter, from online to phone interactions to in-person. Service plays an important role in retail settings and the approach we used herein should be examined further to predict customer satisfaction, customer lifetime value, and impulse purchases. In the hospitality industry, Immersion data could improve guest experiences, ensure personalized service delivery, and foster customer loyalty. In healthcare, neurophysiological metrics could capture clinician engagement and may influence patient outcomes (Rancati and Maggioni, 2023).

There are several limitations to this study. First, the sample size was moderate. While this was addressed by creating a well-matched synthetic dataset, the results may not match those of larger samples. Second, both stores sold high-end clothing to customers directly assisted by sales staff. Greater variation in the type of merchandise and the number of measured sales associates would significantly extend our findings. The protocol here used unintrusive wearables and a commercial software platform that makes such an extension straightforward. Third, the customer base was a nonrepresentative sample of adults living in Southern California who chose to shop at either store. This nonrandom assignment increases ecological validity but reduces generalizability to other demographic segments. Fourth, the study focused on luxury retail, limiting its findings to other retail contexts. Future research could investigate how neurophysiological measures such as Immersion predict purchases in less personalized, or lower-engagement retail environments. Fifth, while the study successfully predicted customer purchases based on sales associate Immersion, future research should explore if physiological responses are associated with customer satisfaction, store loyalty, and customer lifetime value.

The growth of the experience economy (Pine and Gilmore, 2019) has increased the demand for Peak Immersion experiences, which businesses also desire in order to increase customer lifetime value (Diller et al., 2005). Measuring the Immersion of service personnel in restaurants, theaters (Melton et al., 2024), shopping malls, sporting events, and car dealerships could substantially improve the shopping experience and thereby build a base of loyal customers. The Immersion Neuroscience platform displays cleaned data in real time that businesses could monitor while customers shop to improve customer experiences and increase sales. Changes in customer service approaches that seek to increase Peak Immersion and dwell time could also be objectively tested using our methodology. For example, does offering customers refreshments influence Immersion, dwell time, and sales? Or does an unexpected gift during check-out create a neural peak-end experience and increase loyalty (Caruelle et al., 2024)?

The broadest interpretation of the present research is that human beings are functionally social creatures as shown by copious prior research (Brooks, 2012; Zak, 2012; Tomasello, 2014; Zak, 2022). We gregariously congregate for both enjoyment and to fulfill our needs. When one person’s needs coincide with another’s desire to supply what is needed, our neurophysiology synchronizes, creating benefits for those on both sides of a transaction.

## Data Availability

The dataset presented in this study can be found in online repositories. The names of the repository/repositories and accession number(s) can be found at: Open ICPSR openicpsr-203881.
