# Supplementary material for: The contagion of neurologic Immersion predicts retail purchases
Source: Front Neurosci. 2025 Mar 12;19:1533784. doi: 10.3389/fnins.2025.1533784 (PMC11936908; doi:10.3389/fnins.2025.1533784)
Supplement: Supplementary file 1 [file Data_Sheet_1.pdf]

Appendix

The statistical tests in the figures below show that the synthetic data closely match the observed data on means, variances, and correlations. The small variations between synthetic and observed data are due to the the moderate sample size and are typical (Drechsler & Haensch, 2024). The term S_pMSE denotes the synthetic propensity score mean-squared error for Immersion (imm), peak Immersion (peak) and time. S_pMSE is the probability that the synthetic data match the original data, with a lower pMSE indicating a better fit (Snoke et al., 2018).

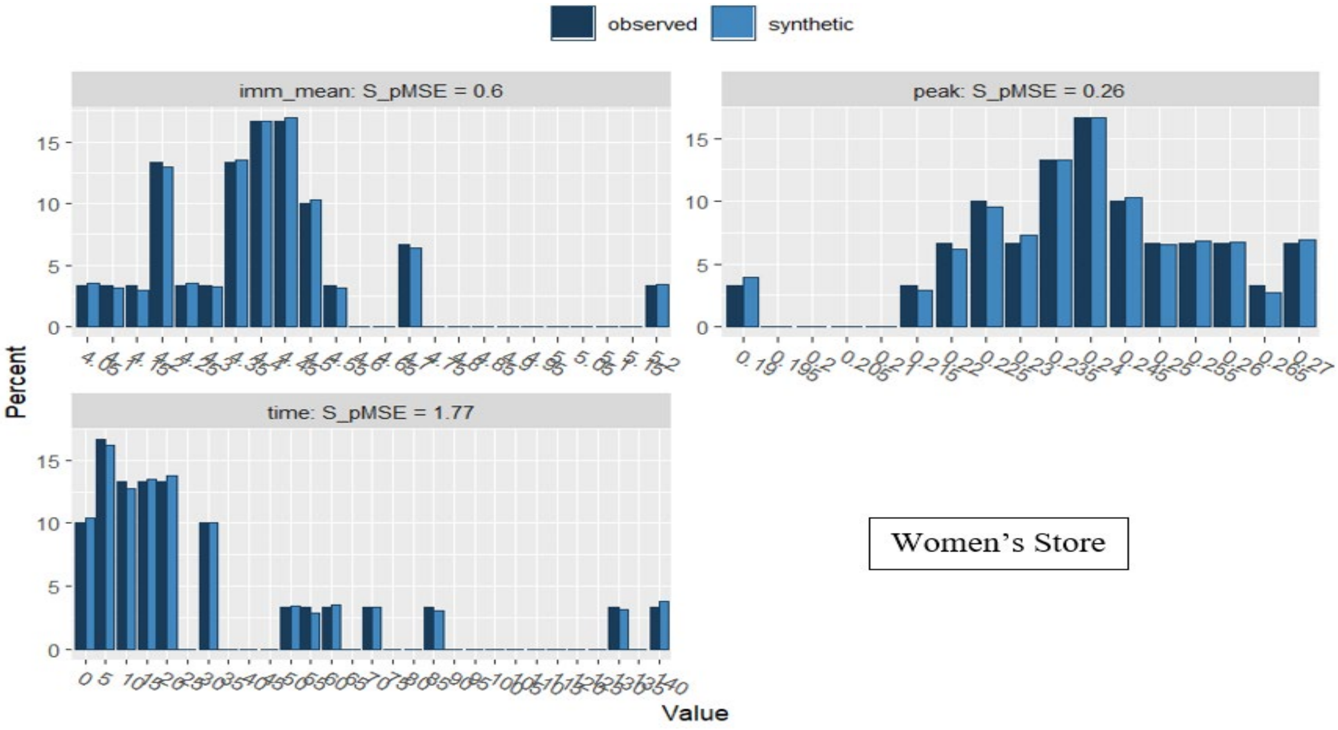

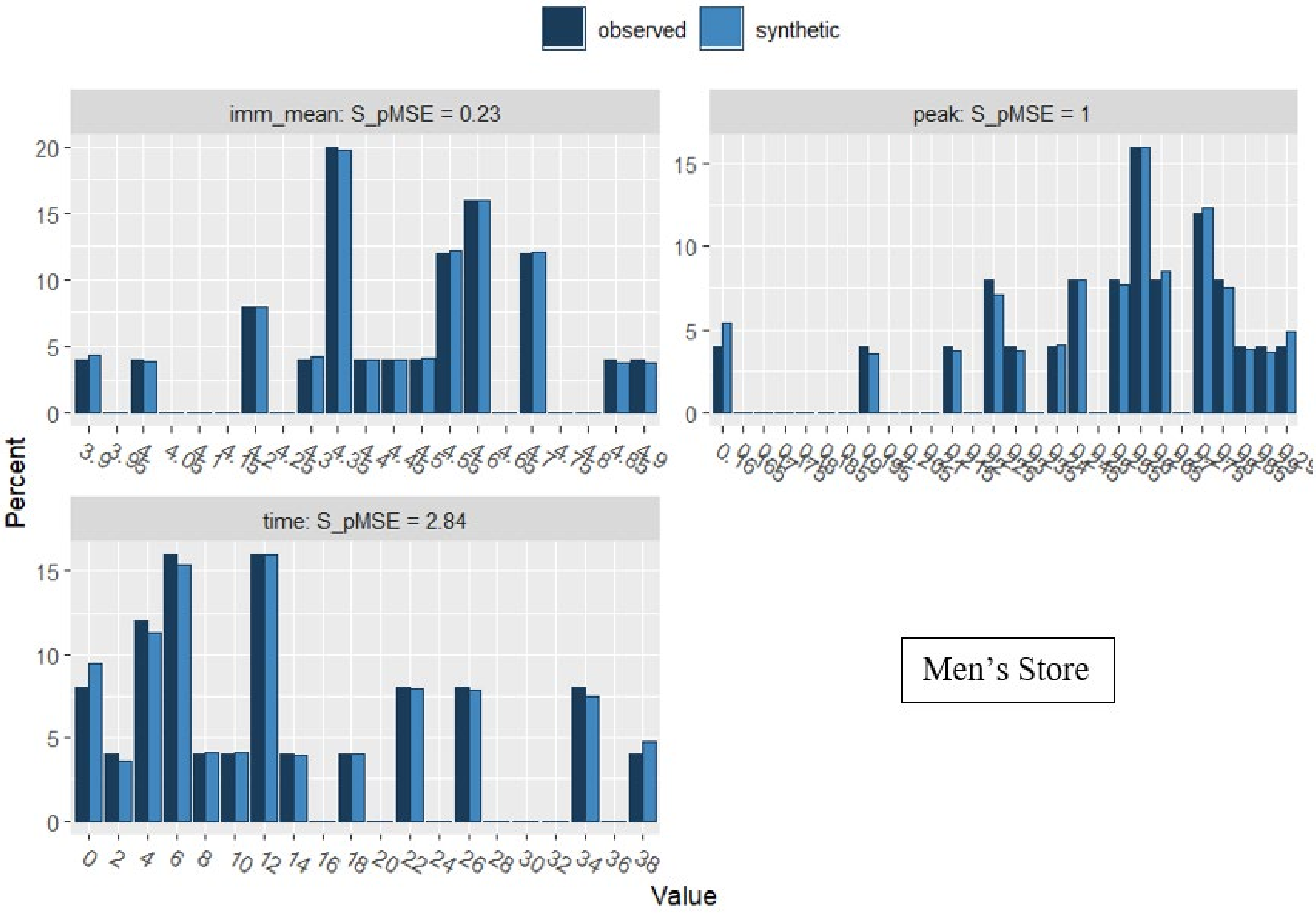

Figures A1-A6: Comparison of the distributions of the original and synthetic data for the women's and men's stores.

Tables A1-A4 report the mediation model regression coefficients and goodness of fit measures for each model identified below the table.

		Coeff.	SE	z value	P(>|z|)	VIF	R^2	F
Women's Store	Indirect Effect	0.775	0.080	9.648	0.000		.101	1125.695
	Direct Effect	3.787	0.242	15.657	0.000		.037	391.745
	Total Effect	4.562	0.230	19.795	0.000	1.113	.047	248.567
Men's Store								
	Indirect Effect	2.059	0.076	27.199	0.000		0.119	1351.90
	Direct Effect	1.036	0.148	7.020	0.000		0.041	428.965
	Total Effect	3.095	0.149	20.714	0.000	1.135	0.175	1065.92

Table A1. Mediation model using synthetic data. Goodness of fit measures and variance inflation factors (VIFs) are all in acceptable ranges.

		Coeff.	SE	z value	P(>|z|)	VIF	R^2	F
Women's Store	Indirect Effect	0.415	0.076	5.439	0.000		0.16	634.8
	Direct Effect	4.120	0.245	16.849	0.000		0.086	313.5
	Total Effect	4.535	0.233	19.463	0.000	1.189	0.089	242.8
	Loyal	0.168	0.008	21.788	0.000	1.155		
	Companion	0.014	0.008	1.703	0.089	1.286		
Men's Store								
	Indirect Effect	2.114	0.077	27.562	0.000		0.162	646
	Direct Effect	1.217	0.150	8.123	0.000		0.046	162.2
	Total Effect	3.331	0.152	21.879	0.000	1.162	0.181	553.6
	Loyal	-0.071	0.009	-7.576	0.000	1.089		
	Companion	0.017	0.010	1.794	0.073	1.105		

Table A2. Mediation model summary using synthetic data and controls. Goodness of fit measures and variance inflation factors (VIFs) are all in acceptable ranges.

		Coeff.	SE	z value	P(>|z|)	VIF	R^2	F
Women's Store	Indirect Effect	1.221	1.427	0.856	0.392		0.048	1.412
	Direct Effect	4.937	4.679	1.055	0.291		0.055	1.622
	Total Effect	6.158	4.672	1.318	0.187	1.05	0.097	1.458
Men's Store								
	Indirect Effect	2.837	1.830	1.550	0.121		0.12	3.155
	Direct Effect	1.312	2.884	0.455	0.649		0.066	1.639
	Total Effect	4.148	3.108	1.335	0.182	1.137	0.293	4.564

Table A3. Mediation model using original data. The men's store mediation model is statistically significant but the women's store is not.

		Coeff.	SE	z value	P(>|z|)	VIF	R^2	F
Women's Store	Indirect Effect	0.574	1.062	0.540	0.589		0.177	1.863
	Direct Effect	5.674	4.688	1.210	0.226		0.209	2.296
	Total Effect	6.248	4.614	1.354	0.176	1.189	0.218	1.738
	Loyal	0.329	0.147	2.245	0.025	1.155		
	Companion	0.038	0.158	0.243	0.808	1.286		
Men's Store								
	Indirect Effect	0.177	1.863	0.177	1.863		0.218	1.952
	Direct Effect	0.209	2.296	0.209	2.296		0.077	0.584
	Total Effect	0.218	1.738	0.218	1.738	1.188	0.314	2.283
	Loyal	-0.138	0.179	-0.769	0.442	1.187		
	Companion	0.042	0.180	0.235	0.814	1.170		

Table A4. Mediation model using original data and controls. Neither the men's store nor the women's store mediation model is statistically significant.
